# Immune checkpoint inhibitor-related encephalitis overlapping with hyperprogression in metastatic lung cancer: a case report

**DOI:** 10.3389/fimmu.2026.1728047

**Published:** 2026-02-26

**Authors:** Weiran Xu, Beihe Cao, Xiaoyan Li

**Affiliations:** Beijing Tiantan Hospital, Capital Medical University, Beijing, China

**Keywords:** encephalitis, hyperprogressive disease, immune checkpoint inhibitors, immune-related adverse events, non-small cell lung cancer

## Abstract

**Background:**

Immune checkpoint inhibitors (ICIs) have significantly improved survival in patients with advanced non-small cell lung cancer (NSCLC), but may rarely trigger severe immune-related adverse events (irAEs).

**Case summary:**

A 64-year-oldpatient with metastatic squamous NSCLC and high programmed death-ligand 1 (PD-L1) expression developed subacute neurological decline following pembrolizumab therapy, concurrent with rapid progression. Cerebrospinal fluid (CSF) analysis revealed inflammatory changes featuring markedly elevated IL-6 levels, while serological and imaging studies excluded infection, supporting a diagnosis of ICI-related encephalitis. Despite neurological symptom resolution with corticosteroids and intravenous immunoglobulin, systemic hyperprogressive disease (HPD) emerged, culminating in fatal tumor progression.

**Conclusion:**

This case underscores the need for vigilance toward the coexistence of rare neurotoxic irAEs and HPD during ICI therapy. Early recognition, multidisciplinary collaboration, and balanced therapeutic strategies are critical to optimizing outcomes.

## Introduction

ICIs have revolutionized therapy for advanced NSCLC, particularly in tumors with high PD-L1 expression. Pembrolizumab, an anti-programmed death-1 (PD-1) monoclonal antibody, is approved as first-line monotherapy in metastatic NSCLC with PD-L1 ≥50%, offering improved survival over chemotherapy in this subgroup (1). We report a case of a patient with metastatic NSCLC who developed immune-related encephalitis concurrent with HPD following PD-1 inhibitor therapy, resulting in a poor clinical outcome despite extensive diagnostic evaluation and treatment efforts. The case highlights the diagnostic challenges and management considerations in such complex scenarios, and we review the relevant literature on immune checkpoint inhibitor (ICI)-related encephalitis and atypical tumor response patterns.

## Case presentation

**Figure 1:**
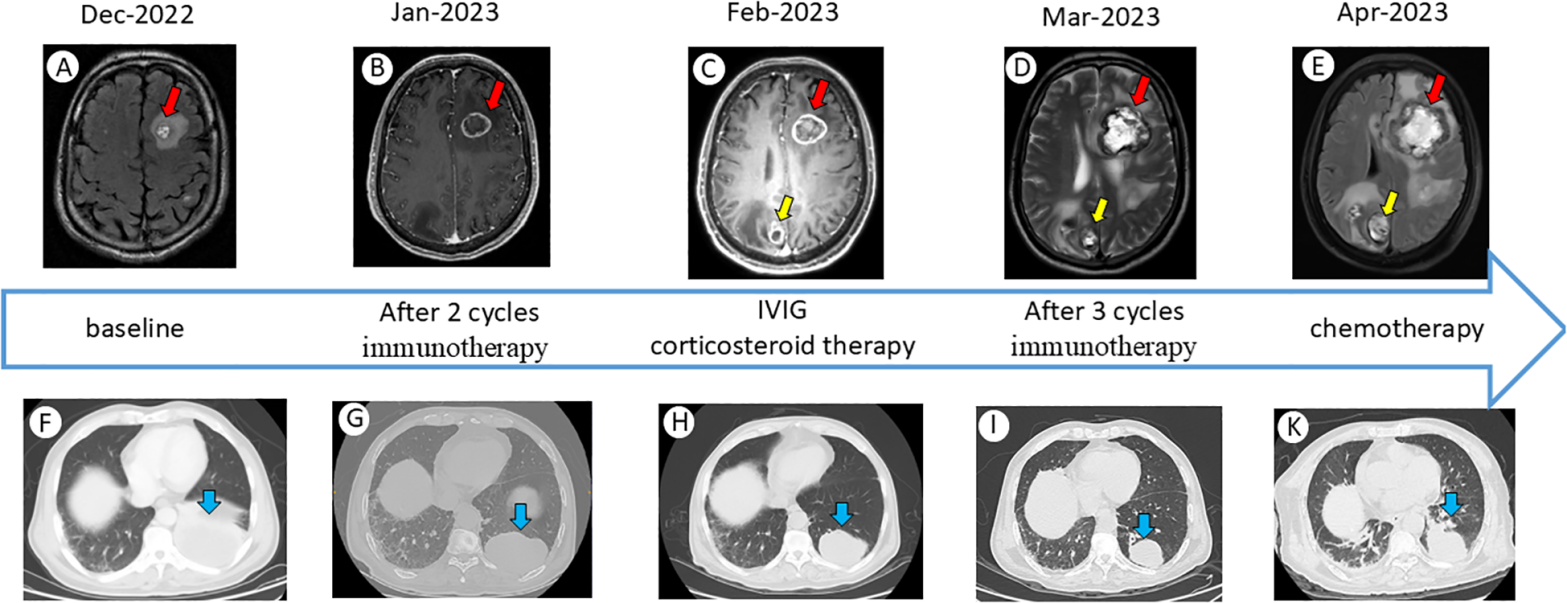
Schematic timeline of temporal evolution in imaging findings. **(A-E)** Dynamic changes in brain MRI over time. Interval growth of a left parietal lobe lesion on serial imaging (red arrow). A newly detected lesion exhibiting sustained growth on serial imaging studies(yellow arrow). **(F-K)** Dynamic changes in chest CT over time. The primary left lower lobe tumor(blue arrow).

A 64-year-oldpatient with a 30 pack-year smoking history presented in October 2022 with cough and left shoulder pain. No family history of hereditary tumors was noted. Imaging revealed a mass in the left lower lobe of the lung and multiple lymph node metastases. Biopsy of the lung mass confirmed squamous cell carcinoma. Staging studies including positron emission tomography-computed tomography (PET-CT) and brain magnetic resonance imaging (MRI) showed widespread metastases: bilateral hilar and mediastinal lymph nodes, multiple bone lesions, and two small brain metastases in the left parietal lobe and right cerebellum (**Figure 1A**). Molecular profiling of the tumor showed a pathogenic TP53 mutation; no actionable driver mutations were detected. The tumor proportion score (TPS) for PD-L1 was 100% by immunohistochemistry. The cancer was staged as cT4N3M1 (Stage IV). Given the high PD-L1 expression and absence of targetable mutations, first-line immunotherapy was planned. He had no significant comorbidities besides well-controlled hypertension, and maintained an Eastern Cooperative Oncology Group (ECOG) performance status score of 1.

In early January 2023, the patient began treatment with pembrolizumab 200 mg intravenously every 3 weeks. He tolerated the first infusion well initially. Two weeks after cycle 1, he developed fever (up to 39 °C) with chills and mild headache. Work-up for infection, including blood cultures and chest imaging, was unremarkable; the fever resolved spontaneously after 48 hours. He received cycle 2 on schedule. Within a week of the second infusion, the patient’s family noted excessive daytime somnolence, intermittent confusion, and memory lapses. He became apathetic and experienced episodes of word-finding difficulty. There was no seizure activity or motor deficit. On examination he was drowsy but arousable, oriented only to person, with slowed speech. Mild neck stiffness was present, though Kernig and Brudzinski signs were equivocal. Cranial nerves were intact and there were no focal neurological deficits aside from slightly slowed limb movements.

**Figure 2:**
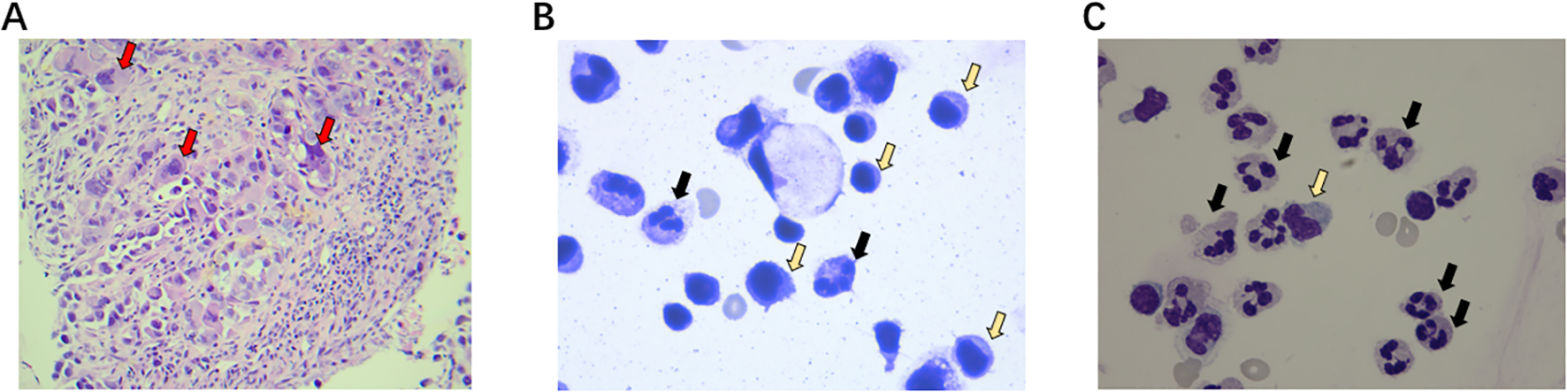
H&E stain from cervical lymph node tumor tissue and cerebrospinal fluid. **(A)** A high density of viable tumor cells (red arrow) with minimal infiltration of tumor-infiltrating lymphocytes. **(B)** Compared with the normal range, the cerebrospinal fluid analysis revealed an increased proportion of neutrophils and a decreased proportion of monocytes. **(C)** After the third cycle of ICI treatment, a more pronounced elevation in the neutrophil percentage was observed in the cerebrospinal fluid, which was characteristic of encephalitis.

Given his cancer history, differential diagnoses included brain metastasis progression, paraneoplastic or autoimmune encephalitis, central nervous system (CNS) infection, and an immune-related adverse event affecting the CNS such as ICI-related encephalitis. An urgent brain MRI was obtained, which revealed that the known brain metastases had increased in size (the larger lesion grew from 18 mm to 24 mm) with surrounding edema; additionally, a new small enhancing lesion was noted in the frontal lobe (**Figure 1B**). These findings were concerning for progression of intracranial metastases, though pseudoprogression could not be excluded. A lumbar puncture was performed. CSF analysis showed an opening pressure of 22 cm H_2_O, white blood cell count of 45/µL (lymphocyte-predominant), protein 85 mg/dL, and glucose 58 mg/dL. Cytology was negative for malignant cells. Extensive infectious studies were negative, including bacterial cultures, cryptococcal antigen, and PCR panels for common viral pathogens. To extensively evaluate for autoimmune and paraneoplastic etiologies, an 18-item autoimmune encephalitis antibody panel was tested in both serum and CSF. The testing was performed using a commercial cell-based assay (CBA) employing double immunofluorescence, without additional rat brain immunohistochemistry. The complete panel returned negative, which included antibodies against: AMPAR1, AMPAR2, CASPR2, GABABR, LGI1, NMDAR, IgLON5, DPPX, GlyR1, D2R, mGluR5, GAD65, GABA_A receptor α1, GABA_A receptor β3, GABA_A receptor γ2, Neurexin-3α, NMDAR2a, and NMDAR2b. Notably, the CSF cytokine analysis demonstrated markedly elevated interleukin-6 (IL-6) and interleukin-8 (IL-8) levels, along with a significantly increased neutrophil proportion in the CSF (**Table 1**, **Figure 2B**), suggesting an inflammatory immune activation in the CNS. Systemic inflammatory markers were also high (serum C-reactive protein peaked at 120 mg/L). No other irAEs were observed. Neurology and infectious disease evaluations suggested possible infection or autoimmune encephalitis. Upon admission to our hospital, the patient received mannitol, dexamethasone, and ceftriaxone, resulting in symptom relief without fever or somnolence. After two cycles of pembrolizumab, imaging demonstrated decreased pulmonary lesions but progressive intracranial lesions, raising suspicion for pseudoprogression.

**Table 1:** Results of cerebrospinal fluid analysis.

Parameters	Reference range	13 Feburary 2023	1 March 2023
IL-6(pg/ml)	0-3.4	155	>1000
IL-8(pg/ml)	0-62	332	500
WBC(/ul) LY% MONO% NEUT%	0-8 60-70 30-40 0-1	45 55 7 35	250 10 8 82
Total protein(mg/dl)	15-45	97.98	77.09
Glucose(mmol/L)	2.5-4.5	3.06	3.49
ADA(U/L)	Not established	1.9	1.5
Cl-(mmol/L)	118-132	122	118

ADA, Adenosine deaminase; WBC, White Bloodcell; LY, Lymphocyte; MONO, Monocyte; NEUT, Neutrophil.

He subsequently received a third cycle of pembrolizumab on February 22, 2023, with continued dexamethasone and mannitol support, remaining asymptomatic during hospitalization. However, from February 28 onward, he developed recurrent fever (peak 39.5 °C), severe fatigue, somnolence, confusion, nausea, vomiting, and anorexia, without respiratory or gastrointestinal symptoms. The patient’s ECOG performance status decreased to 2. The CSF still showed an abnormally high neutrophil percentage on cytologic examination, along with a significant further elevation in cytokine levels, together supporting a diagnosis of immune-related encephalitis (**Table 1**,**Figure 2C**). A multidisciplinary board including oncology, neurology, neuroradiology, and infectious disease specialists convened to formulate a plan. The consensus was to treat presumptively for an immune-related encephalitis, while closely monitoring tumor behavior. High-dose corticosteroid therapy was initiated with intravenous methylprednisolone pulse therapy at a dose of 500 mg daily for 3 days. This was followed by a stepwise tapering regimen of 250 mg daily for 3 days, which was then reduced to 120 mg and subsequently to 60 mg for slow tapering every week. Concurrent intravenous immunoglobulin (IVIG) at 0.4 g/kg/day for 3 days was also administered. Following this intervention, the patient’s level of consciousness and confusion began improving. By the end of the course, he was awake, alert, and cognitively near baseline. His fever and inflammatory markers also abated. To further modulate the autoimmune process, his neurological improvement persisted, and he was discharged home on a slow prednisone taper. This favorable response to immunosuppression strongly supported the diagnosis of ICI-related autoimmune encephalitis, an irAE. At discharge (late February 2023), he had no focal deficits, with only mild short-term memory impairment remaining.

Over the following six weeks, ICI therapy was permanently discontinued due to the severity of the grade 3 neurological immune-related adverse event, aligning with standard clinical guidelines that strongly advise against ICI re-challenge following severe neurotoxicity. During this treatment-free interval, the patient unfortunately experienced marked clinical and radiographic disease progression. Unfortunately, during this period, the patient experienced marked clinical and radiographic disease progression(**Figure 1D**). In April 2023, he developed swelling of the face, neck, and upper limbs, accompanied by prominent chest wall venous distention—clinical signs consistent with superior vena cava syndrome. Contrast-enhanced CT revealed significant enlargement of mediastinal lymph nodes compressing the superior vena cava, along with an increase in the size of the primary left lower lobe tumour (**Figures 1F-K**). The rate of progression was notably accelerated compared to the pre-immunotherapy disease course, consistent with a diagnosis of hyperprogressive disease.

**Figure 3:**
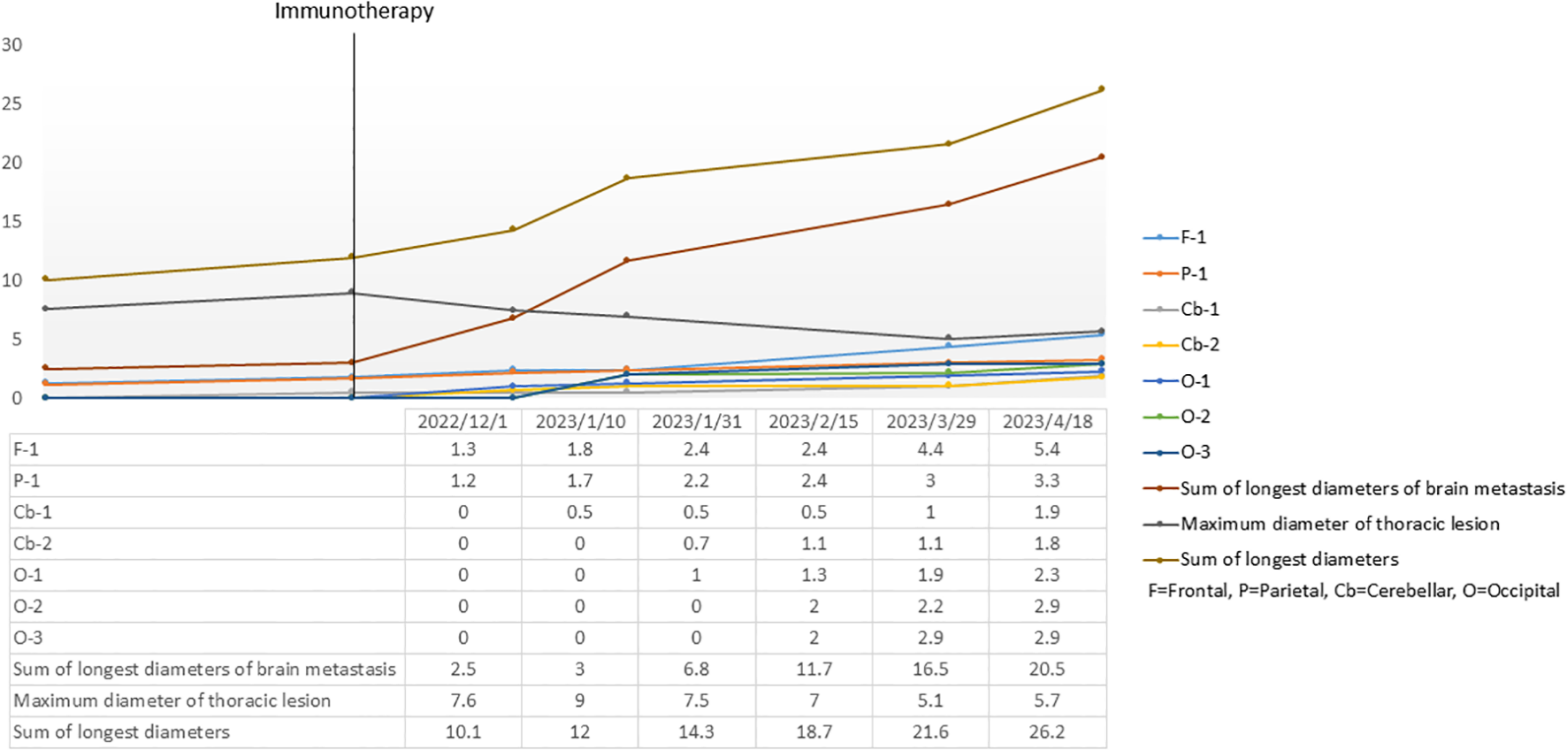
Quantitative assessment of tumor kinetics and evaluation of hyperprogressive disease (HPD). Longitudinal changes in the maximum diameter of key target lesions (thoracic lesion and brain metastasis), illustrating a rapid escalation in tumor burden following the initiation of immunotherapy.

To strictly validate the diagnosis of HPD, we performed a retrospective quantitative analysis of tumor kinetics (**Figure 3**). Measurements of key target lesions, specifically the primary lung mass and brain metastases, demonstrated a drastic deviation from the pre-treatment growth trajectory. The quantitative assessment revealed that the Tumor Growth Rate (TGR) during the immunotherapy interval increased by more than two-fold compared to the baseline kinetics. These standardized quantitative indices provide robust radiological evidence confirming true hyperprogression driven by immune checkpoint blockade, distinct from the natural disease course or pseudoprogression.

On April 21, 2023, salvage chemotherapy was attempted with albumin-bound paclitaxel (100 mg on days 1 and 8) combined with carboplatin (200 mg on days 1 and 8), but the clinical response was limited (**Figure 1E**). A concurrent CT-guided biopsy of the mediastinal lymph nodes was performed, revealing a high density of viable tumour cells with minimal infiltration of tumour-infiltrating lymphocytes, further supporting true tumour progression rather than immune-related pseudoprogression(**Figure 2A**). Given the patient’s rapidly declining performance status, further oncological interventions, including palliative stenting or radiotherapy, were deemed unfeasible. He was transitioned to best supportive care and died from progressive disease in May 2023, approximately five months after initiating immunotherapy.

## Discussion

This case illustrates a distinct clinical phenotype characterized by the concurrent manifestation of two rare immune-dysregulated phenomena—ICI-related encephalitis and HPD — in a patient with metastatic squamous NSCLC. Rather than viewing these as isolated events, the patient’s specific tumor profile, notably the 100% PD-L1 expression, suggests a unique mechanistic overlap driven by immune overactivation.

### Hyper-immune activation in the context of High PD-L1 expression

While ICI-related encephalitis is historically rare, its occurrence in this patient may be mechanistically linked to the extremely high TPS of 100%. We hypothesize that such profound PD-L1 positivity may lower the threshold for fulminant T-cell activation, leading to a “spillover” effect into the central nervous system. This is supported by the CSF findings, which, despite being negative for classic paraneoplastic antibodies, revealed markedly elevated IL-6 and IL-8 levels. Recent evidence posits that pro-inflammatory cytokines like IL-6 function as key mediators in both neurotoxicity and tumor immune evasion. In this specific case, the cytokine storm confined to the CNS likely reflected a systemic dysregulation that paradoxically fueled tumor growth, aligning with the hypothesis that interferon-gamma or cytokine signaling can switch from anti-tumor to pro-tumorigenic in certain genetic contexts.

### The therapeutic dilemma: corticosteroids and HPD acceleration

A critical focal point of this case is the temporal relationship between high-dose immunosuppression and the onset of HPD. The patient received pulse methylprednisolone and IVIG, which successfully reversed the grade 3 neurological toxicity. However, this was immediately followed by an explosive acceleration in tumor kinetics consistent with HPD. However, according to current consensus guidelines, severe neurologic irAEs such as immune-related encephalitis warrant immediate discontinuation of ICIs and initiation of high-dose corticosteroids as first-line treatment, with additional immunomodulatory therapies such as IVIG or plasma exchange in refractory cases (1–3).

While corticosteroids remain essential for managing severe irAEs, emerging evidence suggests that high-dose steroids may attenuate the antitumor immune effects of ICIs, potentially diminishing therapeutic efficacy (4). In particular, early steroid use (within the first 30 days of ICI initiation) has been associated with poorer outcomes in some retrospective analyses (5). Whether corticosteroids directly contributed to hyperprogression in this case remains uncertain, but the temporal association warrants further investigation. In this patient, we postulate a “double-hit” scenario: the initial ICI therapy triggered an aggressive but off-target immune response, and the subsequent high-dose steroids abruptly ablated the nascent anti-tumor immunity without controlling the intrinsic drivers of proliferation, because the *TP53* mutation pathways often implicated in HPD (6). This suggests that in patients with biomarkers predicting high PD-L1 expression, the high-dose steroid protocols for neurotoxicity might catalyze hyperprogression.

### The dilemma of ICI discontinuation and re-challenge

The key point of this case lies in the fulminant tumor progression that ensued immediately after the mandatory discontinuation of pembrolizumab. This brings forth the critical and highly debated topic of ICI re-challenge in clinical practice. While resuming ICI therapy can be a viable strategy to regain tumor control in patients who experience mild to moderate irAEs, it carries a substantial risk of recurrent.

According to current ESMO and ASCO guidelines, permanent discontinuation of ICIs is unequivocally recommended for severe neurological irAEs, such as encephalitis, due to their potential for permanent morbidity or fatal outcomes. Consequently, in our patient, who experienced Grade 3 neurotoxicity, an ICI re-challenge was deemed strictly contraindicated. Coupled with the initiation of high-dose corticosteroids, this created an ideal permissive environment for hyperprogressive disease.

This case heavily underscores the desperate clinical void for patients who achieve potential tumor control but are barred from ICI re-challenge due to severe neurotoxicity. It highlights the urgent need for trials evaluating concurrent targeted immunosuppression that might safely permit ICI re-challenge without reigniting lethal encephalitis.

### Biomarker implications and practical insights

The clinical course highlights the limitation of current monitoring strategies. Interestingly, about half of reported ICI-encephalitis cases have detectable neural-specific autoantibodies such as anti-Ma2, GAD65, and NMDA receptor reflecting an overlap with paraneoplastic neurologic syndromes (7). Our patient had no such antibodies, a scenario consistent with “seronegative” immune encephalitis which is still common in this setting.

Notably, our patient demonstrated markedly elevated CSF and systemic levels of IL-6 and IL-8, both of which are key proinflammatory cytokines implicated in irAEs and tumor immune evasion (8, 9). IL-6 is known to drive T-cell dysregulation and has been associated with resistance to checkpoint blockade as well as enhanced tumor proliferation in various malignancies (10). Recent preclinical and early-phase clinical studies have explored the use of IL-6 or JAK/STAT pathway inhibitors to mitigate severe irAEs while preserving antitumor immunity (11). Targeting IL-8, a neutrophil chemoattractant linked to poor ICI response and hyperprogression, is also under investigation as a novel immunomodulatory approach (12). This suggests that IL-6 and IL-8 levels could serve as early warning signs for this specific HPD-irAE overlap. Consequently, this case suggests that for patients with ultra-high PD-L1 expression presenting with neurotoxicity, a more nuanced therapeutic approach may be required. Future research should investigate alternative steroid-sparing agents, such as IL-6 inhibitors (e.g., tocilizumab) or JAK/STAT pathway inhibitors, which might mitigate severe irAEs like encephalitis without providing the immunosuppressive environment that facilitates hyperprogression. Furthermore, when high-dose steroids are unavoidable, we recommend shortening the interval for restaging imaging to detect potential steroid-associated hyperprogression immediately.

## Conclusion

We described a patient with metastatic PD-L1-high squamous NSCLC who developed ICI-related encephalitis overlapping with hyperprogressive disease. The case highlights the diagnostic and therapeutic challenges when severe irAEs mimic or coincide with cancer progression. ICI-related encephalitis, while rare, should be suspected in patients on immunotherapy who present with unexplained neurologic symptoms, and managed emergently with immunosuppressive therapy. Especially for patients with ultra-high PD-L1 expression, clinicians should maintain a low threshold for neurological workup within the first 6 weeks of therapy.

In parallel, early tumor restaging is crucial to identify atypical response patterns such as pseudoprogression or hyperprogression. This case emphasizes the importance of a comprehensive approach to immunotherapy patients: one that rapidly addresses irAEs without losing sight of tumor control. Close monitoring, flexibility in treatment strategy, and multidisciplinary expertise were key in our patient’s care. Implementing routine screening for CSF IL-6, IL-8 prior to ICI initiation may help identify patients at risk for the HPD-irAE overlap. In cases where severe neurotoxicity requires high-dose steroids, concurrent restaging imaging should be performed at shorter intervals to detect potential steroid-associated hyperprogression early. Future research should investigate alternative steroid-sparing agents, such as IL-6 inhibitors, to manage severe irAEs without compromising tumor control in this high-risk subgroup.

As immunotherapies become increasingly utilized, awareness of these challenges and continued research into predictive markers are paramount to improving patient outcomes.

## Patient perspective

The patient’s family members acknowledged the inherent risks of treatment but expressed profound concern over the rapid clinical deterioration. Written informed consent was obtained prior to treatment initiation.

## Data Availability

The original contributions presented in the study are included in the article/supplementary material. Further inquiries can be directed to the corresponding author.
