# Correction: Immune checkpoint inhibitor-related encephalitis overlapping with hyperprogression in metastatic lung cancer: a case report

**DOI:** 10.3389/fimmu.2026.1840228

**Published:** 2026-04-16

**Authors:** Weiran Xu, Beihe Cao, Xiaoyan Li

**Affiliations:** Beijing Tiantan Hospital, Capital Medical University, Beijing, China

**Keywords:** encephalitis, hyperprogressive disease, immune checkpoint inhibitors, immune-related adverse events, non-small cell lung cancer

The funder “Beijing Natural Science Foundation, 7242007” to Xiaoyan Li and “Beijing Hospitals Authority Clinical medicine Development of Special Funding support, YGLX202517” to Xiaoyan Li were erroneously omitted.

The original version of this article has been updated.

